# The whole is greater than the sum of its parts: profiles of multiple mental health risk factors using Latent class analysis

**DOI:** 10.1186/s13034-021-00380-8

**Published:** 2021-06-14

**Authors:** Kristin Göbel, Caroline Cohrdes

**Affiliations:** 1 Department of Epidemiology and Health Monitoring, Mental Health Unit, Robert Koch Institute, General-Pape-Straße 62-66, 12101 Berlin, Germany; 2 Present Address: Department of Educational Science and Psychology, Division of Developmental Science and Applied Developmental Psychology, Freie Universität Berlin, Habelschwerdter Allee 45, 14195 Berlin, Germany

**Keywords:** Mental health, Youth, Multiple risk factors, Latent class analysis, Depression

## Abstract

**Background:**

The exposure to an accumulation of various risk factors during childhood and adolescence relative to a single risk is associated with poorer mental health. Identification of distinct constellations of risk factors is an essential step towards the development of effective prevention strategies of mental disorders. A Latent class analysis (LCA) extracts different combinations of risk factors or subgroups and examines the association between profiles of multiple risk and mental health outcomes.

**Methods:**

The current study used longitudinal survey data (KiGGS) of 10,853 German children, adolescents and young adults. The LCA included 27 robust risk and protective factors across multiple domains for mental health.

**Results:**

The LCA identified four subgroups of individuals with different risk profiles: a *basic-risk (51.4%), high-risk (23.4%), parental-risk (11.8%) and social-risk class (13.4%).* Multiple risk factors of the family domain, in particular family instability were associated with negative mental health outcomes (e.g. mental health problems, depression, ADHD) and predominately comprised late adolescent girls. The social environment represented a more common risk domain for young males.

**Conclusion:**

The understanding of multiple risk and different risk “profiles” helps to understand and adjust targeted interventions with a focus on vulnerable groups.

## Background

Mental health problems originate early in life, affect 10–20% of children and adolescents worldwide, and can have a long-lasting effect throughout life [1]. Empirical evidence shows that a number of mental health problems during childhood tend to continue or predict other problems in adulthood [2]. Attention deficit/hyperactivity disorder (ADHD) is one of the most common mental disorders in childhood and adolescence, while major depression is more common during young adulthood. Both illnesses are related to an enormous disease burden for individual and society [3, 4]. Hence a better understanding of the factors associated with mental health during childhood and adolescence is of great importance.

The presence of a single risk factor during childhood and adolescence is very common and associated with little to no developmental consequences [5, 6]. According to the multiple risk perspective, the exposure to an accumulation of various risk factors relative to a single risk is associated with poorer mental health in children and adolescents, and described as a cause for mental disorders in young people [2, 5, 7–9]. The absence or presence of multiple risk factors explains why some children living in a single parent household may be adjusted well (i.e. absence of multiple risks) and why some children with both parents may show developmental delays (i.e. presence of multiple negative events).

Fortunately, not all children who are exposed to multiple risk factors experience mental health problems as the presence of protective factors are equally relevant. Research found evidence that protective factors in various domains (e.g. personal, familial and social resources) buffered the adverse impact of multiple risk factors [5]. Protective factors contribute to a resilient development by strengthening children’s mental health when being exposed to risks [9]. Furthermore, childhood and adolescence do not occur in isolation but rather within multiple domains, including the family, the school and the environment [1–3]. Multiple risk and protective factors can cumulate across those different domains. For instance, children living in a single parent household may receive significant social support as a buffer for maladjustment. With other words, the significance of any one aspect of individual development becomes meaningful when taking other aspects of the environment into account [10]. The nature and interplay of those factors across domains hinder or contribute to the development of mentally healthy individuals [2]. Albeit literature shows how each context can contribute to specific outcomes, far less is known about the multilevel impact regarding risk factors within diverse contexts. The purpose of the present study was to identify the subgroups of co-occurrence of multiple risk factors across domains in a young sample and their association with mental health outcomes.

### Risk factors for mental health

Innumerous investigations of risk and protective factors for childhood and adolescence mental health exist and are commonly separated into three domains: individual, familial and social [9]. At the level of the individual, children’s own characteristics like temperament [5, 11], self-esteem [12] or self-efficacy can have an effect on their mental health. For instance, high self-efficacy is the optimistic belief in one’s own ability to be able to cope and adapt to difficult demands in life or stressful events, and has been associated with fewer adjustment problems [13]. Moreover, biological factors such as low birthweight (LBW) as well as prenatal exposure to alcohol and tobacco [14, 15], duration of breastfeeding [14, 16], chronic illnesses [17] and postnatal problems (e.g. adjustment or infections) [18, 19] have shown to contribute to negative mental health outcomes like developmental delays and behavioural disorders [6].

Apart from individual risk factors, familial factors are among the most significant predictors of negative mental health outcomes. Children’s first contact with their environment begins with their parents who significantly contribute to their socialization of appropriate behaviour from infancy and beyond [20]. The quality of parenting has important consequences for a child’s health outcomes [21]. Children exhibit less emotional and behavioural dysfunctioning if raised in authoritative homes with warmth, autonomy and clear rules [22]. For instance, Cohrdes and Göbel (submitted) examined a sample of 4,258 German adolescents with an age range of 11–17 years showing that parenting characterized by warmth was related to fewer behavioural problems, while pressure was related to a higher risk of behavioural and emotional problems. Likewise, a change in family structure for instance a transition from a two-parent to a single-parent family due to divorce is associated with more behavior and emotional problems [23]. Additionally, numerous studies have shown associations between several family factors and negative mental health outcomes, for instance teenage mothers [5, 24], large family size [25], low parental education [5, 26], low parental health [27], high parental stress [28] and parental psychopathology [29–31].

Furthermore, an accumulating body of research worldwide has focused on the complex, insidious problem of child maltreatment which is repeatedly linked to adverse mental and physical health. Child maltreatment involving physical or sexual abuse have been extensively studied as risk factors for adverse long-term outcomes, however also psychological abuse or neglect, has received increasing attention. Precisely, emotional neglect or abuse consists of behaviours towards children such as rejection, isolation, or verbal assault and have serious adverse effects on mental health [32–34].

In addition to risk factors originating within the family, the surrounding or social environment nearby exerts influences on children. For instance, as adolescents become increasingly independent, they begin to seek out peer relationships as source of intimacy and social support. Peer relationships offer support while exploring different roles or promoting self-disclosure [35]. Adverse social relations and experiences enhance the risk for mental disorders in youth [11]. For instance, a large body of research has shown that experiences with discrimination [36] or being teased by peers or family members are associated with a wide range of psychosocial problems, including aggressive behavior, anxiety, and depression [37]. In general, positive social networks contribute to the development of a child by encouraging coping and offering support [9, 35].

Substantial research has shown that multiple risk factors portend maladjustment in children and adolescents, thereby making an impact on the developmental course across adolescence and emerging adulthood [1–3].

Identification of distinct constellations of risk factors is an essential step to tackle differences in children’s development over the life course and towards the development of effective prevention strategies of mental disorders [4, 5].

### Empirical approach towards multiple risk factors

A general approach towards the understanding of multiple risk factors and child development used by several researchers is a variable-oriented method such as a cumulative risk index [9].

By using the sum of dichotomized risk factors to construct a cumulative risk index, Rutter [8] marked the beginnings of subsequent numerous investigations demonstrating the associations between multiple risks and less adaptive mental health outcomes [9, 20, 38]. For instance, Wille et al. [9] examined children’s mental health and the number of potential risk and protective factors using a representative sub-sample of 2863 families with children and adolescents aged 7–17 years. Main findings suggest that the co-occurrence of risk factors affect mental health problems significantly. Parental health-related quality of life and conflicts within the family showed the strongest association with a child’s mental health problems.

However, the cumulative risk index emphasizes two disadvantages. The approach treats multiple risk factors equally by giving each of them the same weight. Furthermore, multiple risk factors are substitutable, by which the pure exposure matters rather than the nature of the risk factors [39]. Moreover, an index does not provide enough information about the interaction between risk factors and its variation across distinct subgroups [39].

One complementary approach to the variable-oriented method is a person-centered approach such as the Latent class analysis (LCA). A LCA categorizes individuals into homogenous subgroups (or classes) within a heterogeneous population according to certain characteristics or patterns [40]. A person-centered approach can provide important insight regarding the interaction of the individual’s entire range of risk factors and its association to less adaptive mental health outcomes [41]. In other words, the demonstration of different combinations of risk factors across multiple domains (i.e. individual, family, and social environment) provides a better understanding of the associations between risk factors and aversive mental health outcomes [39]. Despite the growing popularity of person-centered approaches as, several studies used LCA in different contexts (e.g. [42, 43, 44]), it has less well been demonstrated with regard to multiple risk factor for mental health across several domains. Previous studies have almost exclusively focused on single domains or cluster analysis as person-centered statistical technique. Amongst previous research, only two studies applied a person-centered method for modelling multiple mental health risks. Parra, DuBois, and Sher [45] identified four subgroups of adolescents (7th and 11th graders) with distinct profiles of risk factors (i.e. low risk, socioeconomic disadvantage, peer high risk, and family high risk class) from different contextual domains (i.e., individual, family, peer, school, and neighborhood) with predicted levels of depressive symptoms and conduct problems both concurrently and over time.

Lanza and colleagues [39] extracted four unique profiles with 13 risk factors across child, family, school, and neighborhood domains for preschool children (N = 750). Results showed that the highest risk for negative outcomes are subgroups characterized by several risks across several domains.

However, several shortcomings were noted for these studies, for instance, (1) a restricted choice of risk factors (i.e. robust risk factors for mental health were not assessed); (2) no parent-report data were used (i.e. multi-informant data for a comprehensive assessment of youth functioning was not considered); and (3) no representative population or a small sample with a restricted age range was used for analyses.

In spite of those two studies using LCA to identify risk profiles with multiple risk factors across several domains, the topic is still insufficiently explored.

### The current study

The focus of the current study was primarily on identifying homogeneous subgroups of adolescents according to their exposure to a broad range of mental health risk indicators with a large sample using a person-centered approach (e.g. LCA). We expected to find at least four subgroups of multiple risk combinations concurrent with previous research [39, 45]. Furthermore, we hypothesized to identify subgroups that are not only distinct due to the number of risks (e.g. low, medium vs. high risk) as inferred by a variable-centered approach but also due to the nature of the risk factors (e.g. subgroup with family-related risks).

The second aim was to examine the extracted homogeneous subgroups and their associations with different mental health issues or disorders (i.e. internalizing and externalizing mental health problems, reported diagnoses of ADHD and depression). We hypothesized that subgroups characterized by multiple risks will show a high probability for negative mental health outcomes. Furthermore, we also expected subgroups characterized by specific risks to show different combinations of internalizing and externalizing mental health problems, reported diagnoses of ADHD and depression. Additionally, as reported by Fanti and Henrich [46], we expect externalizing problems more often to be found in early adolescent boys, and internalizing problems more prevalent in late adolescent girls.

The current study improves upon prior research by including key constructs known to be robust risk factors for adolescent psychopathology from multiple domains (i.e. individual, family, and social); using multiple informant information (i.e. self and parental reports); and lastly by conducting analyses with data from a large, national, and longitudinal German sample of children and adolescents.

The identification of distinct subgroups according to their multiple risk exposure and their relation to mental health outcomes can help to better identify vulnerable individuals with a special need and specific strategies of prevention and intervention.

## Methods

### Sample

The German Health Interview and Examination Survey for Children and Adolescents (KiGGS) is part of the federal health monitoring framework established at the Robert Koch- Institute. KiGGS incorporates several cross-sectional surveys (i.e. KiGGS Baseline, KiGGS Wave 1 and KiGGS Wave 2) with all waves collapsing to a longitudinal component (KiGGS Cohort). The current study reports on children, adolescents and young adults from the KiGGS Cohort, who across an eleven-year period completed at least two out of the three assessments (i.e. KiGGS Baseline and Wave 2). Two different publications describe the study design, sample information and attrition in more detail [47, 48].

In brief, starting in 2003, 0–17 year olds were recruited from 167 sample points across Germany providing information on a wide spectrum of health-relevant topics related to the child and the primary caregiver. Two follow-ups were initiated approximately 6 and 11 years later (KiGGS Wave 1: 2009–2012, KiGGS Wave 2: 2014–2017), obtaining wide-ranging, reliable data on child and adolescent health from birth to childhood and from adolescence to young adulthood. The final sample for this study (KiGGS Cohort) included data from 10,853 children, adolescents and young adults and their primary caregiver with an age range between 10 to 31 years (mean age 19.1, SD = 5.2) and 44.6% females.

### Measures

#### Indicators

The KiGGS Cohort study comprises a broad set of determinants including risk and protective factors for child and adolescent mental health. In line with previous research, several robust risk and protective factors for mental health were collected across three domains: individual (e.g. chronic illness, self-efficacy), familial (e.g. family cohesion, maltreatment), and social (e.g. social support, peer relation). Table 1 provides an overview of 27 selected indicators, corresponding measurement instruments, operationalization and assessment period. While some indictors were based on self-constructed scales or items (e.g. parental illness, parental attention), others originated from standardized and valid measures with acceptable reliability (e.g. self-efficacy, chronic illness). Time-independent indicators (e.g. birth weight, prenatal exposure to smoking/drinking) and retrospective indicators (e.g. abuse/neglect, parental illness) have been answered only once while other information is available for at least two assessment periods (e.g. self-efficacy, social support). Information available at more than one measurement point was collapsed to form new indicators based on the time-variant nature (e.g. family instability) or to strengthen the indicator by using a within-person mean across several assessment periods (see Table 1, scale description for further information).

**Table 1:** List of risk factor for mental health from the KiGGS studies

Risk factor	Instrument	Reference	Item/Scale Description	Time period	Report
Individual level					
Low birth weight	Self-constructed		Low birth weight < 2500 g (1), else (0)	T0	Parent
Postnatal problems	Self-constructed		One or more problems like infection, adjustment (1), else (0)	T0	Parent
Breastfeeding	Self-constructed		Partly to fully (1), never (0)	T0	Parent
Chronic illness	Mini European Health Module (MEHM)	[79]	One or more illness like asthma, epilepsy or diabetes (1), else (0)	T0, T1	Parent
Self-efficacy	General Self-efficacy Scale (GSE)	[80]	Within-person mean < 25% of distribution of sum scores (1), else (0)	T0, T1, T2	Parent and Self
Family level					
Parental education	Comparative Analysis of Social Mobility in Industrial Nations classification (CASMIN)	[81]	Low education qualification (1), else (0) based on T0 or if missing T2	T0, T2	Parent
Parental severe illness	Self-constructed		In case of stroke, heart attack or cancer (1), else (0)	T2	Self
Parental well-being	Personal Well-being index (PWI-A)	[82]	Sum score < 25% of distribution; low (1), else (0)	T2	Parent
Parental unemployment	Self-constructed		Present (1), else (0)	T0, T2	Parent
Prenatal exposure to alcohol and tabacco	Self-constructed		Smoking or drinking during pregnancy (1), else (0)	T0	Parent
Teenage mother	Self-constructed		Maternal age < 18 (1), else (0)	T0	Parent
Parental divorce	Self-constructed		Parental divorce (1), else (0)	T2	Parent
Family instability	Self-constructed		Not living with both parents at one of the three measurement times (1), else (0)	T0, T1, T2	Parent and Self
Parental attention	Self-constructed		Frequency of a selection of eight joint activities such as playing, singing, or sports. Sum score < 25% of distribution; low (1) else (0)	T0	Parent
Family size	Self-constructed		Number of siblings > = 2 (1), else (0)	T2	Self
Parental stress		Adapted from [83]	Covering parental stressors e.g. family demands, household requirements, child-related stress, conflicts with current or former spouse or life partner, compatibility of family and work, and financial strain Sum score < 25% of distribution; high (1), else (0)	T2	Parent
Family cohesion	Family climate scales (FKS)	[84]	Within-person mean < 25% of distribution; low (1), else (0)	T0, T1, T2	Parent and Self
Family dysfunction	Adapted from the Adverse Childhood Experiences International Questionnaire (ACE-IQ)	[85]	Either parent incarceration, drug/alcohol addiction, mental illness/suicide attempt of a household member (1), none (0)	T2	Parent
Parenting: warmth and support	Zurich Short Inventory for Parenting Behavior (ZKE)	[86]	Sum score < 25% of distribution; low (1), else (0)	T2	Parent and Self
Parenting: psychological pressure	Zurich Short Inventory for Parenting Behavior (ZKE)	[86]	Sum score > 25% of distribution; high (1), else (0)	T2	Parent and Self
Physical neglect or abuse	Childhood trauma Questionnaire (CTQ)	[87]	Never (0), sometimes to very often (1)	T2	Self
Emotional neglect or abuse	Childhood trauma Questionnaire (CTQ)	[87]	Never (0), sometimes to very often (1)	T2	Self
Sexual abuse	Childhood trauma Questionnaire (CTQ)	[87]	Never (0), sometimes to very often (1)	T2	Self
Social level					
Well-being with peers	KINDL-R, Kidscreen-27	[88]	Within-person mean < 25% of distribution of standardized scores (1) else (0)	T0, T2	Parent and Self
Well-being in school	KINDL-R, Kidscreen-27	[88]	Within-person mean < 25% of distribution of standardized scores (1) else (0)	T0, T2	Parent and Self
Perceived social support	Social support scale		Within-person mean < 25% of distribution (1) else (0)	T0, T1, T2	Parent and Self
Discrimination experiences	Adapted from German Youth Institute (DJI) Foreigners Survey	[89]	Any discrimination experience averaged across eight domains (i.e., race, gender, weight) sometimes to very often (1), never	T2	Self

Furthermore, this study used information answered by parents (e.g. parental education status), along with combined information from parental reports for their children (age < 11 years) and self-reports from adolescents and young adults (age > 10 years), e.g., well-being in school and with peers. As not all risk factors are inherently dichotomous, the use of a cut point at the 75th percentile with 25% of the distribution towards a less favourable score changed risk variables into groups of absence and presence of risk. The approach to use binary variables was taken to facilitate the interpretation of latent classes and reduce the number of parameters estimated by the models [49].

#### Mental health outcomes

The KiGGS Cohort study provided self- and parent-reported diagnoses of mental disorders, such as ADHD and depression. Parent and self-reported data of lifetime ADHD diagnoses given by a physician or psychologists were assessed at KiGGS Wave 2. Lifetime diagnoses of depression were reported by young adults (age > 18 years) at KiGGS Wave 2. Mental health problems were assessed using a parent and self-reported Strengths and Difficulties Questionnaire (SDQ, [50]) at KiGGS Wave 2. Parents and children or adolescents answered several questions assessing symptoms on four subscales: conduct problems, emotional problems, hyperactivity, and peer problems within the last 12 months. The difficulties score for each subscale was calculated and banding scores were used to categorise into ‘normal’ vs. ‘borderline / abnormal’ scores [51, 52].

Moreover, as suggested by Goodman, Lamping, & Ploubidis [50], the SDQ allows a differentiation of mental health problems into internalizing and externalizing problems. While internalizing behaviours (subscales emotional and peer problems) are described as social-emotional problems directed inwards related to social withdrawal, depression and anxiety, inversely externalizing behaviours (subscales conduct problems and hyperactivity) are described as behavioural problems directed outwards like hyperactivity, non-compliance, or aggression [53].

### Statistical analysis

#### Latent class analysis (LCA)

LCA was applied using Mplus 7.1 to relate a set of observed indicators to the optimal number of subgroups with similar risk behavior profiles [54]. A set of 27 binary risk indicators for children’s mental health infers latent class membership. The LCA processes almost 135 million possible patterns of unique responses for the given risk indicators into meaningful subgroups reflecting dominant profiles in the sample. A comprehensive overview of LCA can be found in Collins and Lanza [40].

In a first step, successive LCA models with ascending number of classes were examined and model selection was determined based on underlying statistical evidence and theoretical assumptions [55]. Subsequently, the appropriate number of latent classes relayed on several statistical criteria, including the Akaike information criterion (AIC), Bayesian information criterion (BIC), and the sample size-adjusted Bayesian criterion (aBIC). In addition, the entropy and posterior probabilities were examined for each model. To determine the number of classes in latent class analysis, the Lo-Mendell-Rubin test (LMRT) and the bootstrapped likelihood (BLRT) are used and provide a data-driven way to evaluate the relative adequacy of a (K-1)-class model compared to a K-class model. Missing data for the risk and protective factors varied between 0.46% (family instability) and 5.80% (postnatal problems). The missing rate for each variable was on average below 5% and, was addressed by using full information maximum likelihood (FIML) under the assumption of “missing at random” (MAR) available in Mplus.

#### Multinomial logistic regression

The last step builds upon the LCA “three-step” procedure by which following the model building process and the assignment to the latent classes or groups, a multinomial logistic regression model was estimated to include covariates (i.e. age and gender) and mental health outcomes (i.e. internalizing and externalizing mental health problems, diagnoses of ADHD and depression) to examine differences in the probabilities of each subgroup to be at risk for distinct mental health problems (for more information see [56]).

## Results

### Descriptive statistics

Distribution of risk factors in the sample population varies widely. While some risk factors are less pronounced in the sample, for instance teenage mothers (0.31%) or low parental attention (1.47%), others seem very common such as discrimination experiences (45.33%) or a dysfunctional family life (54.80%) (see Table 2).

**Table 2:** Prevalence rates for each risk factor (N = 10,853)

Risk factors	%	n
Individual level		
Low Birth Weight	5.51	583
Early infant problems	20.50	2096
Breastfeeding	19.04	2029
Chronic illness	24.70	2681
Self-efficacy	25.88	2726
Family level		
Parental education	12.68	1367
Parental severe illness	35.93	3900
Parental well-being	23.28	1037
Parental unemployment	14.11	1531
Prenatal smoking or drinking	25.60	2719
Teenage mother	0.31	33
Parental divorce	12.20	548
Family instability	22.95	2479
Parental attention	1.47	160
Large family size	17.15	1861
Parental stress	28.34	1250
Family cohesion	30.74	3296
Family dysfunction	54.80	5947
Parenting: warmth and support	22.76	982
Parenting: psychological pressure	28.16	1215
Physical neglect or abuse	21.68	1317
Emotional neglect or abuse	31.23	1897
Sexual abuse	5.26	320
Social level		
Well-being with peers	27.06	2878
Well-being in school	24.90	2609
Perceived social support	21.95	2335
Discrimination experiences	45.33	4920

A cumulative score of risk factors shows that 1.65% of individuals (mean age = 19, SD = 4.33) in the sample endorsed no risk, while 0.09% of individuals (mean age = 20, SD = 4.32) reported fifteen or more risk factors. On average, individuals reported four to five risk factors (mean = 4.90, SD = 2.67) of mental health over a period of 11 years.

### Model selection and number of classes

Mplus computed models with one through seven latent classes with multiple set of starting values. Table 3 shows fit indices for all classes. The six-class and seven-class model were not identified well with no sufficient replication of best log-likelihood value. Results suggest that models with four or five latent classes could be considered based on LMRT and BLRT p values. The model quality based on the entropy showed little difference between solutions slightly favouring the 5-class solution, however the posterior probabilities for the class membership was below 0.80 [57]. Moreover, the additional fifth class for the five-class model did not add more interpretive value compared to the four-class model. Based on the quality of the model, the interpretability of the latent classes, and the parsimony principle, we selected the four-class model as most optimal.

**Table 3:** Summary of model fit indices of successive latent class models (N = 10,853)

Model	AIC	BIC	aBIC	Entropy	LMRT p value		BLRT p value
Two-class	235,638.280	236,053.935	235,872.797	0.618	0.000		0.000
Three-class	232,624.262	233,251.391	232,978.094	0.704	0.000		0.000
**Four-class**	**231,236.097**	**232,074.700**	**231,709.245**	**0.750**	**0.000**		**0.000**
Five-class	230,389.630	231,439.706	230,982.093	0.763	0.000		0.000
Six-class^**a**^	229,846.766	231,108.316	230,558.545	0.725	0.000		0.000
Seven-class^**a**^	229,480.724	230,953.747	230,311.818	0.667	0.000		0.000

*AIC* Akaike information criteria, *BIC* Bayesian information criteria, *aBIC* adjusted BIC; *LMR LTR p value* Lo-Mendell-Rubin likelihood ratio test p value, *BLRT LRT p* Bootstrapped Likelihood ratio test p value

Best-fitting model is indicated in bold

^a^Latent class solution not well identified

**Fig. 1:**
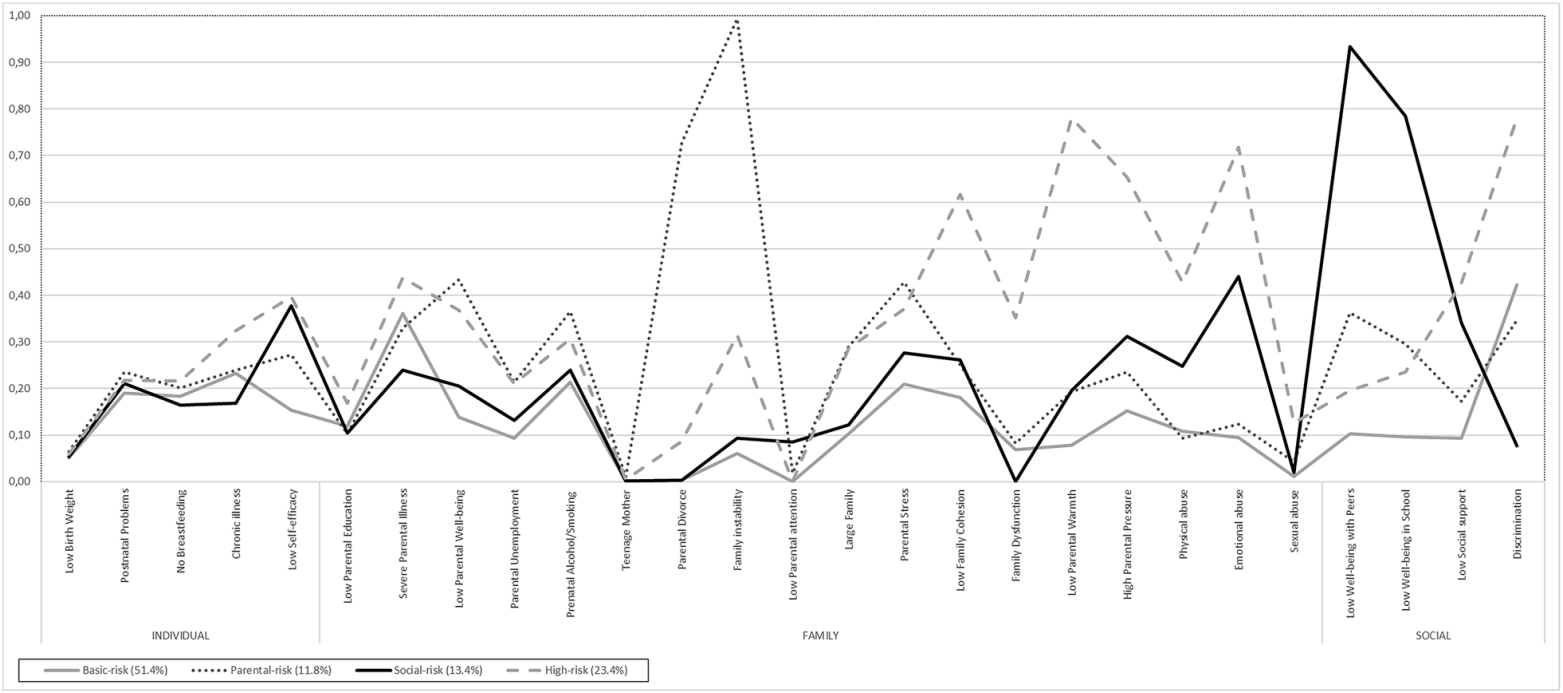
Item probability for each latent class. The horizontal axis displays the 27 risk factors for mental health, while the vertical axis show the item probabilities of the four-class solution

Figure 1 shows the probabilities of reporting each risk factor by class.

In general, few reported risk factors showed similar probabilities across classes (i.e. birth problems, LBW, breastfeeding and teenage mother) and therefore have little influence on class differentiation. Other risk factors, on the contrary, were more predominant by showing high variance regarding probability across classes. Precisely, most risk factors from the individual domain were less pronounced to differentiate classes compared to risk factors from the family and social domain.

Table 4 shows conditional probabilities of indicators of latent classes. All reported indicators below show significant differences among latent classes.

**Table 4:** Conditional probabilities of indicators by latent class

Indicators	Basic-risk (51.4%)	Parental-risk (11.8%)	Social-risk (13.4%)	High-risk (23.4%)
Individual level				
Low Birth Weight	0.051	0.065	0.054	0.059
Postnatal Problems	0.191	0.235	0.211	0.218
No Breastfeeding	0.183	0.201	0.165	0.216
Chronic illness	0.233	0.24	0.168	0.324
Low Self-efficacy	0.154	0.272	0.378	0.396
Family level				
Low Parental Education	0.119	0.103	0.104	0.168
Severe Parental Illness	0.361	0.330	0.240	0.438
Low Parental Well-being	0.138	0.433	0.205	0.368
Parental Unemployment	0.094	0.213	0.132	0.211
Prenatal Alcohol/Smoking	0.213	0.365	0.239	0.306
Teenage Mother	0.001	0.010	0.002	0.005
Parental Divorce	0.003	0.727	0.003	0.087
Family instability	0.060	0.993	0.093	0.315
Low Parental attention	0.001	0.018	0.085	0.000
Large Family	0.103	0.290	0.122	0.286
Parental Stress	0.209	0.428	0.277	0.371
Low Family Cohesion	0.181	0.251	0.262	0.617
Family Dysfunction	0.069	0.082	0.000	0.351
Low Parental Warmth	0.079	0.193	0.196	0.781
High Parental Pressure	0.152	0.235	0.312	0.653
Physical neglect/abuse	0.108	0.093	0.248	0.428
Emotional neglect/abuse	0.095	0.124	0.44	0.718
Sexual abuse	0.012	0.043	0.02	0.126
Social level				
Low Well-being with Peers	0.103	0.362	0.933	0.196
Low Well-being in School	0.096	0.296	0.784	0.236
Low Social support	0.093	0.172	0.341	0.427
Discrimination	0.422	0.346	0.077	0.781

Class 1 (*basic-risk*) is composed of a high proportion of individuals (51.4%) who are likely to report very few risk factors. Compared with members of the other classes, individuals in this class were less likely to report low self-efficacy (15.4%), low parental well-being (13.8%), parental unemployment (9.4%) or stress (20.9%), and insufficient parenting (high pressure, less warmth). Additionally, members show more social support (9.3%) and well-being with peers (10.3%) and within school (9.6%).

About half of all risk factors (n = 14) were reported by less than 10% of individuals with that class membership.

Class 2 (*parental-risk*) is composed of 11.8% of individuals with few reports on individual or social risk factors but with a focus on parent-related risks. Compared to other classes, almost all members (99.3%) reported an unstable family structure (i.e. not living with both parents within the study period) and 72.7% of individuals experienced a parental divorce. Additionally, the class is characterized by the highest probability of parents reporting low well-being (43.3%), parental stress (42.8%) and prenatal exposure to alcohol and smoking (36.5%).

Class 3 (*social-risk*) included individuals (13.4%) likely to report risk factors from the social domain. Precisely, most class members showed a very low well-being with peers (93.3%) and within school (78.4%). Additionally, the class is composed of second highest proportion of individuals reporting high parental pressure (31.2%), physical (24.8%) and emotional (44%) abuse or neglect.

Class 4 (*high-risk*) is the second largest class with 23.4%. Class membership is characterized by individuals likely to report a large number of risk factors (n = 11) across all domains compared to the other classes. Most class members reported low family cohesion, high family dysfunction, insufficient parenting (i.e. low warmth und high pressure), child maltreatment (i.e. emotional neglect/abuse, physical neglect/abuse and sexual abuse) and experiences with discrimination experience. Additionally, some individuals reported chronic illnesses (32.4%), low parental education (16.8%) and severe parental illnesses (43.8%).

### Latent class membership prediction

Table 5 shows prevalence rates and confidence intervals for age, gender and mental health outcomes within classes. Covariates (i.e. age, gender) and mental health outcomes (i.e. diagnoses of depression, ADHD, internalizing and externalizing mental health problems) showed significant associations with class membership (see Table 6). In general, *social*-risk class members are younger with more males compared to the other risk classes. In turn, *high-risk* class members are more likely to be older and female compared to the other classes. Compared to the *basic-risk* members, the *parental-risk, social-risk* and the *high-risk* class members had higher odds of having internalizing and externalizing mental health problems, depression and ADHD. Compared to the *high-risk* class members, *social*-*risk* class members showed lower odds for externalizing problems. Additionally, the *parental-risk* class showed lower odds for externalizing problems compared to the *high-risk* class and lower odds for depression diagnoses compared to the *high-risk* and *social-risk* class.

**Table 5:** Prevalence rates and confidence intervals for age, gender and mental health outcomes

	Basic-risk (51.4%)	Parental-risk (11.8%)	Social-risk (13.4%)	High-risk (23.4%)
Age^a^	20 (4.72)	18 (4.99)	13 (3.46)	22 (4.44)
Gender (female)	53.5 (52.2, 54.8)	53.6 (50.8, 56.3)	47.9 (45.4, 50.5)	56.1 (54.2, 58.0)
Internalizing problems	22.2 (21.2, 23.4)	39.6 (36.9, 42.3)	44.2 (41.6, 46.7)	38.4 (36.5, 40.3)
Depression diagnosis	2.8 (2.4, 3.3)	2.7 (1.9, 3.8)	0.7 (0.4, 1.3)	13.3 (12.1, 14.7)
Externalizing problems	19.2 (18.2, 20.3)	34.5 (31.9, 37.1)	41.1 (38.6, 43.6)	32.2 (30.4, 34.1)
ADHD diagnosis	4.2 (3.7, 4.8)	8.6 (7.2, 10.3)	6.9 (5.7, 8.3)	9.4 (8.3, 10.5)

^a^Mean (std), KiGGS Wave 2

**Table 6:** Results of the multinomial logistic regression predicting latent class membership (N = 10,853)

Covariates/ Outcomes	Parental vs. Basic			Social vs. Basic			Risk vs. Basic			Parental vs. Risk			Social vs. Risk			Parental vs. Social		
	RRR	95% CI	*p* value	RRR	95% CI	*p* value	RRR	95% CI	*p* value	RRR	95% CI	*p* value	RRR	95% CI	*p* value	RRR	95% CI	*p* value
Age^**a**^	0.94	0.93–0.96	0.000	0.65	0.63–0.67	0.000	1.09	1.09–1.11	0.000	0.86	0.84–0.87	0.000	0.59	0.57–0.61	0.000	1.44	1.40–1.48	0.000
Gender	1.05	0.93–1.19	0.401	0.82	0.71–0.94	0.005	1.11	1.00–1.23	0.042	0.95	0.82–1.09	0.482	0.74	0.63–0.86	0.000	1.28	1.09–1.51	0.003
Internalizing problems	1.81	1.58–2.08	0.000	1.86	1.62–2.14	0.000	2.07	1.85–2.32	0.000	0.87	0.75–1.01	0.077	0.89	0.76–1.05	0.187	0.97	0.82–1.14	0.744
Depression diagnosis	1.27	0.87–1.85	0.207	3.11	1.63–5.91	0.001	3.97	3.23–4.87	0.000	0.31	0.22–0.46	0.000	0.78	0.41–1.47	0.449	0.40	0.20–0.82	0.013
Externalizing problems	1.57	1.36–1.81	0.000	1.38	1.19–1.59	0.000	2.08	1.84–2.35	0.000	0.75	0.64–0.88	0.001	0.66	0.56–0.78	0.000	1.13	0.95–1.35	0.151
ADHD diagnosis	1.57	1.22–2.01	0.000	1.30	0.98–1.72	0.065	1.52	1.24–1.87	0.000	1.02	0.79–1.32	0.844	0.85	0.63–1.14	0.295	1.20	0.88–1.64	0.243

*RRR* adjusted relative risk ratio, *CI* confidence interval

^a^In years

## Discussion

This research investigated different profiles of multiple risk factors for mental health and their associations to less adaptive mental health outcomes.

To complement the general approach towards multiple risk factors using a variable-oriented method (i.e. cumulative risk index), a person-centered method was used to provide a clearer picture regarding the interplay of an individual’s array of risk factors for mental health. The Latent class analysis (LCA) demonstrated different constellations of 27 risk factors across an individual, familial and social domain and their prediction of mental health problems, depression, and ADHD. In line with previous research and our prediction, a set of four risk classes was identified as optimal out of millions of possible combinations. Precisely, the analysis extracted classes not only characterized by the number of risk factors (e.g. low, medium, high) but also identified risk profiles based on very specific risk factor constellations. While a multiple risk index considers only the mere exposure of several risks, this person-centered approach highlights the specific nature of the risk factors and provides information about the interaction between risk factors and its variation across distinct subgroups.

### Differences between risk classes and associations with mental health outcomes

In general, some risk factors showed small impact regarding the distinction between risk classes. For instance, individual risk factors (e.g. breastfeeding, LBW, postnatal problems) were less likely to be reported by the respondents. Albeit previous research has shown their relevance and contribution to negative mental health outcomes like developmental delays and behavioural disorders in children and adolescents [14, 18], individual factors are less dominant compared to adverse family-specific risks when it comes to children’s mental health [9]. Results confirm the crucial role of family-specific risk factors across the extracted classes.

The largest class (*basic-risk* class; 51.4%) showed a low profile of risk factors for mental health with two minor exceptions. More than 30% of class members stated to have experienced discrimination of some sort (e.g. appearance, weight) and reported a parent with severe illness (i.e. stroke, cancer, heart attack). As these findings correspond with the relatively high prevalence rates of discrimination or teasing from other national adolescent samples (e.g., Canada 25.7%, [58]; US 24.3%, [59]) and cardiovascular diseases such as stroke or heart attack evolved into the most prevalent adult illnesses and leading causes of death worldwide [60], the relatively high prevalence rates in the *basic-risk* class is not outstanding.

Furthermore*, basic-risk* class members are far less likely to show any kind of mental health problem or disorder (i.e. depression and ADHD) compared to the other risk classes. This may be related to the finding that *basic-risk* class members endorse other factors, which could help to compensate the negative effect of discrimination and distress about parental health, such as high self-efficacy, positive parenting (i.e. high amount of warmth and less pressure), high family cohesion and social support.

On the contrary, the other risk classes (i.e. *high-risk, parental-risk* and *social-risk*) do show less individuals endorsing factors, which could help to buffer negative effects, for instance social support, well-being in school and with peers. In general, *high-risk* class members are older and more likely to be female; moreover, they reported the highest number of risk factors among classes, particularly in the family domain. For instance, insufficient parenting (less parental warmth and high pressure) and child maltreatment are more prominent among members of the *high-risk* class. These results support previous research by highlighting the significance of the family life for mental health [9], as those risk class members also show a greater likelihood for mental health problems (i.e. internalizing and externalizing problems) and mental disorders (i.e., depression and ADHD) as compared to the other classes. Furthermore, discrimination (especially towards appearance, weight and gender) is reported by a large number of individuals (78.1%) within the *high-risk* class. As females, who perceive more pressure regarding appearance and are more often victims of teasing by peers and family compared to males [61] are overrepresented in the *high-risk* class, the higher prevalence of individuals reporting discrimination is comprehensible. Weight-based teasing in adolescence from family members and peers seems to be a crucial factor for low self-esteem, emotional distress and depression [61, 62]. Additionally, a number of existing studies provided evidence that a history of abuse predicted increasing depressive symptoms only for females as they are more likely to cope with stress via an avoidant (or ruminative) coping style [63].

A multitude of studies exists with a focus on adversities, which are directly threatening and harmful for a child’s development, for instance child maltreatment and violence [32]. Nevertheless, instability and unpredictability within the family life may also be unfavourable and damaging [64]. Family instability or the change in the family structure by divorce, remarriage, cohabitation, and union dissolution results in ambiguity and insecurity regarding boundries, goals, values and roles within the family [65]. Those changes are associated with an accumulation of stressors for parents and children, and moreover may provoke anxieties for children that persist into adulthood [66, 67]. There are claims stating that parental divorce is one of the most stressful event during childhood with long lasting consequences [68].

Almost all individuals with a class membership in the smallest group (*parental-risk*) reported family instability and parental divorce. Additionally, class members showed an elevated risk for internalizing and externalizing problem behaviour and ADHD. Those findings are in line with previous research claiming that family instability is associated with negative cognitive and behavioural outcomes [69, 70]. For this reason, it may be beneficial to present selective intervention strategies to improve resilience and coping strategies by directly working with children of divorce (e.g. [71, 72]). Mental health professionals are advised to work closely with the educational sectors such as primary care or schools to improve early detection of risk factors from infancy to childhood to adolescence, to provide treatment and to prevent manifestation of mental disorders.

The members of the *social-risk* class showed very specific risk factors in the social domain—well-being in school and with peers. However, as compared to the other risk classes, *social-risk* class members are younger and more likely to be males. Peer-related bullying behaviour or victimization as well as school-related conflicts may explain the difference between classes. For example, in the present study peer-related well-being comprises items of helping and relying on each other and school-related well-being comprises items of getting along well with teachers or being satisfied in school. In general, boys report significantly more often being bullied compared to girls, moreover self-reported victimization declines with increasing age [73]. Younger children are more likely to report peer victimization as compared to older individuals. Additionally, research suggests that boys are more likely than girls to experience teacher-related conflict and disciplinary action in school [74, 75]. Compared to the *basic-risk* class, all negative health outcomes (i.e. internalizing and externalizing problems and diagnoses of depression or ADHD) are more likely for the *social-risk* class members. Precisely, being a member of the* social-risk* class triples the risk of being diagnosed with depression compared to the *basic-risk* group after controlling for age and gender. Several studies confirm the association of well-being in school or with peers on adverse mental health consequences [76, 77].

A few evidence-based and evaluated preventive intervention programmes proved to reduce bullying and relational aggression. For instance, the fairplayer.manual is a multicomponent preventive intervention programme with an emphasis on the group mechanisms of bullying, and therefore, intervening, at the school class level (for seventh to ninth graders) and is designed to address high-risk-group children or causal risk factors, respectively [78]. However, more efforts should be directed towards the implementation of such programmes to improve mental health of children and adolescence and prevent mental disorders, for instance focusing on nationwide rather than regional implementation.

While the study furthers the general understanding of the interaction between multiple risk factors from several domains and its influence on developmental health outcomes, certain limitations should be noted. First, some relevant risk-related variables were assessed retrospectively, elevating the risk for recall bias especially for information like breastfeeding routine or postnatal problems (e.g. excessive crying). Second, associations between risk classes and mental health outcomes cannot be considered as causal, but rather as a connection or mutual relationship between variables. Third, while most variables, like gender and lifetime prevalence, are inherently categorical, several continuous risk factors were dichotomized according to a cut-off by scoring the upper forth of the distribution and thus only represent rough estimators.

Finally, no predictions regarding transitions between classes or developmental trajectories are possible and may constitute the object of future studies. For instance, as life-time risk for certain factors increase with age and risk exposure changes with time, longitudinal measures of risk patterns would be a worthwhile future direction (e.g. using latent transition analysis).

## Conclusions

Substantial research has shown that the exposure to an accumulation of various risk factors (called multiple risk perspective) relative to a single risk is associated with poorer mental health. Moreover, multiple risk research has commonly involved variable-centered approaches such as cumulative risk index. Although the approach demonstrated that a specific number of risk factors makes individuals more vulnerable towards negative mental health outcomes, results of a cumulative risk index does not help in targeting particular ecological domains (e.g. individual, familial, social) for prevention, intervention and health policies. The topic is of great importance, as children’s functioning within the family and the peer environment have long-term developmental consequences for health, well-being, and achievement across adolescence and adulthood [45]. To complement this approach and to improve further understanding of the interplay between multiple risk factors, a person-centered method (e.g. LCA) was introduced. LCA allows for the identification of heterogeneous groups of individuals who share a particular combination of risk factors, which may help to better identify special treatment needs according to specific risk constellations. While an array of 27 risk factors seemed overwhelming in terms of preventive approaches, a four-class solution generated by the LCA provides a parsimonious and concise view of the interplay between those numerous risk factors and aids towards diverse prevention strategies. The understanding of multiple risk and different risk “profiles” helps to adjust interventions and its treatment specification by focusing on a particular vulnerable group, such as late adolescent girls with problems within their family or young males with difficulties in school and with peers. Furthermore, recent research shows that an effective prediction of children with greatest risk for aversive mental health may help to direct resources for intervention measures more successfully.

## Data Availability

Not applicable.
